# Towards trustworthy seizure onset detection using workflow notes

**DOI:** 10.1038/s41746-024-01008-9

**Published:** 2024-02-21

**Authors:** Khaled Saab, Siyi Tang, Mohamed Taha, Christopher Lee-Messer, Christopher Ré, Daniel L. Rubin

**Affiliations:** 1 Department of Electrical Engineering, Stanford University, Stanford, CA USA; 2 Department of Neurology, Stanford University, Stanford, CA USA; 3 Department of Child Neurology, Stanford University, Stanford, CA USA; 4 Department of Computer Science, Stanford University, Stanford, CA USA; 5 Department of Biomedical Data Science, Radiology, and Medicine, Stanford University, Stanford, CA USA

**Keywords:** Epilepsy, Electroencephalography - EEG

## Abstract

A major barrier to deploying healthcare AI is trustworthiness. One form of trustworthiness is a model’s robustness across subgroups: while models may exhibit expert-level performance on aggregate metrics, they often rely on non-causal features, leading to errors in hidden subgroups. To take a step closer towards trustworthy seizure onset detection from EEG, we propose to leverage annotations that are produced by healthcare personnel in routine clinical workflows—which we refer to as workflow notes—that include multiple event descriptions beyond seizures. Using workflow notes, we first show that by scaling training data to 68,920 EEG hours, seizure onset detection performance significantly improves by 12.3 AUROC (Area Under the Receiver Operating Characteristic) points compared to relying on smaller training sets with gold-standard labels. Second, we reveal that our binary seizure onset detection model underperforms on clinically relevant subgroups (e.g., up to a margin of 6.5 AUROC points between pediatrics and adults), while having significantly higher FPRs (False Positive Rates) on EEG clips showing non-epileptiform abnormalities (+19 FPR points). To improve model robustness to hidden subgroups, we train a multilabel model that classifies 26 attributes other than seizures (e.g., spikes and movement artifacts) and significantly improve overall performance (+5.9 AUROC points) while greatly improving performance among subgroups (up to +8.3 AUROC points) and decreasing false positives on non-epileptiform abnormalities (by 8 FPR points). Finally, we find that our multilabel model improves clinical utility (false positives per 24 EEG hours) by a factor of 2×.

## Introduction

The scalp electroencephalogram (EEG) is a non-invasive and valuable technique to measure the brain’s electrical activity. Unlike other modalities that image the brain (e.g., fMRI, PET), EEG enables continuous analysis of rapid changes in the brain’s electrical activity. In the intensive care unit (ICU), EEG is critical for the detection of seizures that may lack a behavioral correlate and worsen brain injury. Moreover, EEG is an essential tool to diagnose and care for epileptic patients of all ages^1^.

While analyzing EEG data is a critical healthcare task, it poses several challenges. First, the continuous recording of hours of multi-channel EEG results in a vast amount of data that requires thorough interpretation, which is a highly time-consuming and costly task that demands deep neurologic-epileptologic understanding. Second, the gold-standard for EEG analysis is done by fellowship-trained clinical neurophysiologists, who have not only been trained to identify seizure patterns, but also many common artifacts. For example, common artifacts on EEG signals may include muscle movement or environment noise, along with countless non-epileptiform abnormalities such as spikes and slowing. Finally, there is a shortage of EEG specialists, and as a result, low resource communities lack access to EEG interpretation^2^. Thus, there is a strong need to develop reliable tools that help clinicians analyze EEG data more efficiently.

Many studies have shown that deep learning (DL) techniques present great promise for automated seizure detection. There have been substantial efforts for curating large and publicly available EEG datasets, such as the Temple University Hospital Seizure Detection (TUSZ) corpus that includes thousands of EEGs from hundreds of patients^3,4^. The availability of large public datasets has enabled rapid progress in benchmarking and improved seizure detection models^5–10^. Recently, a DL model named SParCNet was trained on 6097 EEGs from 2711 patients, annotated independently by 20 fellowship-trained neurophysiologists, and was found to match or exceed most experts in classifying seizures^11^.

Due to the high-stakes nature of healthcare, trustworthiness of DL models remains a major roadblock to clinical adoption^12,13^. Building trust requires addressing multiple facets, including model interpretability, transparency, and robustness across subgroups. Interpretability refers to the ability to explain why predictions are made, transparency involves techniques like analyzing model representations and decision boundaries to provide global understanding of model behavior^14,15^, and robustness across subgroups refers to reliable performance across diverse inputs like different patient groups and disease types. As a first step towards trustworthy seizure onset detection, this work focuses on robustness by investigating performance across clinically relevant subgroups. Alarmingly, there has been a growing body of work revealing that healthcare models with “expert-level” performance often rely on non-generalizable features^16,17^, resulting in unexpected drops in performance over hidden subgroups^18,19^ or under data distribution shifts^20^. While many studies report impressive overall seizure detection performance^6,11^, such studies lack the in-depth analysis needed to understand the clinically meaningful failure modes of existing models. For example, pediatric EEGs look drastically different from adult EEGs, different seizure types display unique EEG patterns, and there may be different types of abnormalities present in EEGs recorded from the ICU as compared to other clinical settings^1^; as a result, models may underperform on specific age groups, seizure subtypes, or ICU patients. Unfortunately, conducting an in-depth error analysis requires manual interpretation of both EEGs and model predictions over a diverse set of studies, making it a costly process. However, a clear understanding of a model’s systematic errors is critical to provide trust in model predictions for clinical adoption.

In this work, we provide a strategy to scale training data, conduct a subgroup robustness analysis, and improve the trustworthiness of seizure onset detection models in a cost-effective manner. As opposed to relying on expensive gold-standard labels, which require a fellow-trained neurophysiologist to label EEGs outside existing clinical workflows, we propose to leverage seizure annotations that are produced by healthcare personnel within existing clinical workflows^5^—which we refer to as workflow notes. Since workflow notes are produced as part of routine clinical practice, we are able to train our DL models on an unprecedented scale of 68,920 EEG hours. To conduct an in-depth error analysis we stratify the evaluation set of EEG recordings into clinically relevant subgroups and analyze discrepancies in seizure onset detection performance in each subgroup. In particular, we use a combination of patient metadata (e.g., age), expert-provided subgroup labels (e.g., seizure types), along with numerous EEG attributes, such as spikes, slowing, movements, jerks, photoelectric stimulation, hyperventilation, and more (full list in Supplementary Table 3), that are readily available from workflow notes.

To improve model robustness to non-epileptiform abnormalities and hidden subgroups, we utilize the workflow notes to increase class specificity. Specifically, as opposed to training a binary classification model (seizure or no seizure onset), we train a multilabel model to classify 25 classes in addition to seizure onset, such as spikes, slowing, and hyperventilation. In addition, we study how our improvements in seizure onset detection robustness translate to clinical utility by tracking the false positives per 24 h for different deployment settings.

## Results

### Results overview

**Fig. 1:**
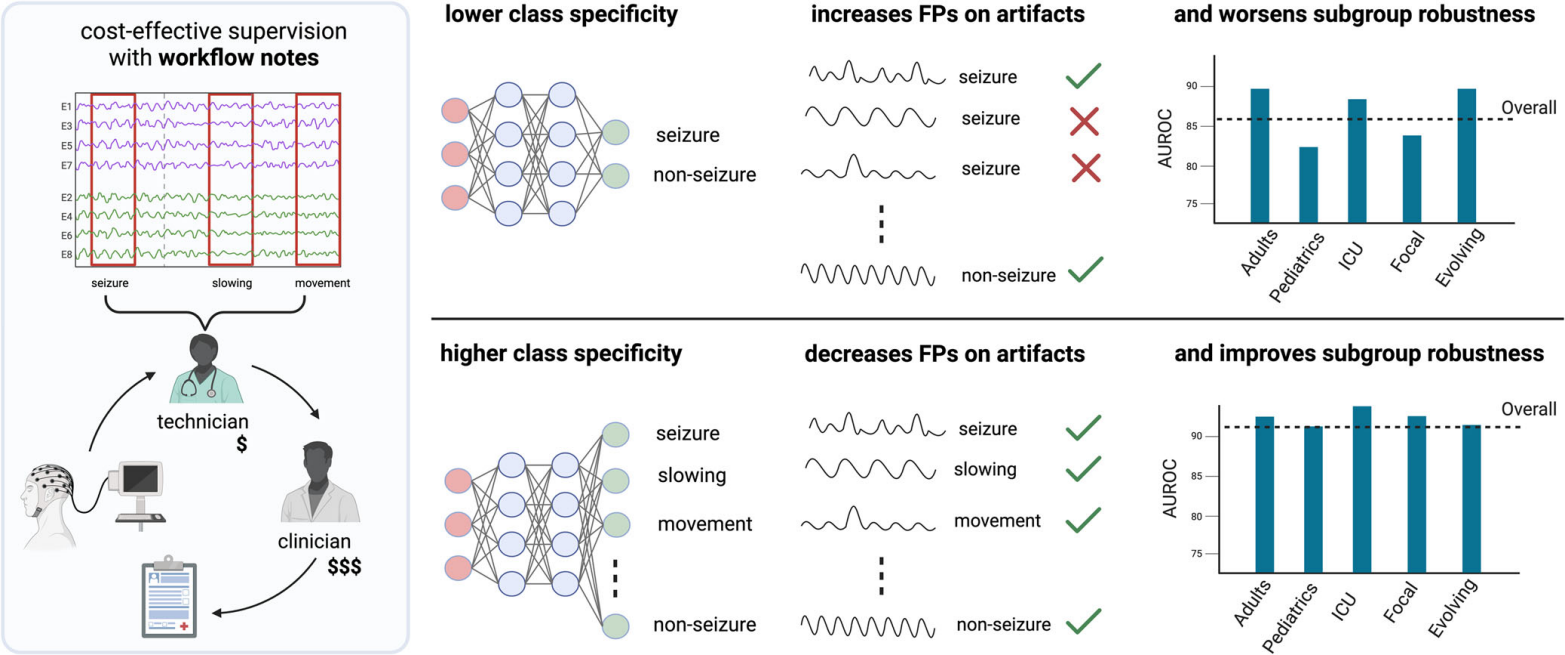
Results overview. We find that increasing class specificity by providing additional supervision decreases false positives on artifacts and improves subgroup robustness. Importantly, we supervise our models on large scale data (68,920 EEG hours) using readily available notes produced within clinical workflows (left panel).

We first describe how we utilize workflow notes to scale supervision to 68,920 EEG hours (4,135,225 60-s EEG clips) in a cost-effective manner, and show that training a model to detect seizure onset using workflow notes greatly improves performance compared with a model trained with a smaller set of gold-standard, expert-labeled EEG clips. We further utilize the workflow notes to reveal that even with large-scale training, our binary seizure onset detection model underperforms on clinically relevant subgroups of patients, and has higher false positive rates for non-seizure EEG clips with abnormal patterns. To improve our model’s performance across subgroups, we train a multilabel model to classify 25 attributes extracted from the workflow notes, in addition to seizure onset (Fig. 1). Finally, we propose a metric of clinical utility to assess the degree to which the multilabel model improves clinical utility over a range of settings.

### Scaling training data with workflow notes

Following previous studies^5,21^, our task of interest is to classify the existence of a seizure onset in a 60-s EEG clip. Each EEG contains 19 electrodes that sample voltage readings at 200 Hz, therefore the input to the model is a 60-s EEG clip $x\in {{\mathbb{R}}}^{12,000\times 19}$ and the output is a binary label *y* ∈ {0, 1} indicating the existence of a seizure onset in that clip. To evaluate and compare the performance of deep learning models on the task of seizure-onset detection, we curated a gold-standard evaluation set of 626 EEG hours (37,588 60-s EEG clips) labeled by two fellowship-trained EEG readers.

Since acquiring gold-standard labels for all 68,920 hours of EEG (or 4,135,225 clips) would be extremely costly, we used a cost-effective technique that leverages workflow notes proposed by Saab et al.^5^. As illustrated in Fig. 1, EEG monitoring in clinical settings involves an initial, collaborative analysis of the EEG signal by a diverse team, consisting of technicians, fellows, students, and board-certified epileptologists. Using the facilities of the clinical EEG acquisition system (Nihon Kohden), preliminary annotators mark potential seizures, abnormalities, and artifacts, which serve as a reference for a board-certified clinician’s subsequent analysis and final diagnosis.

**Fig. 2:**
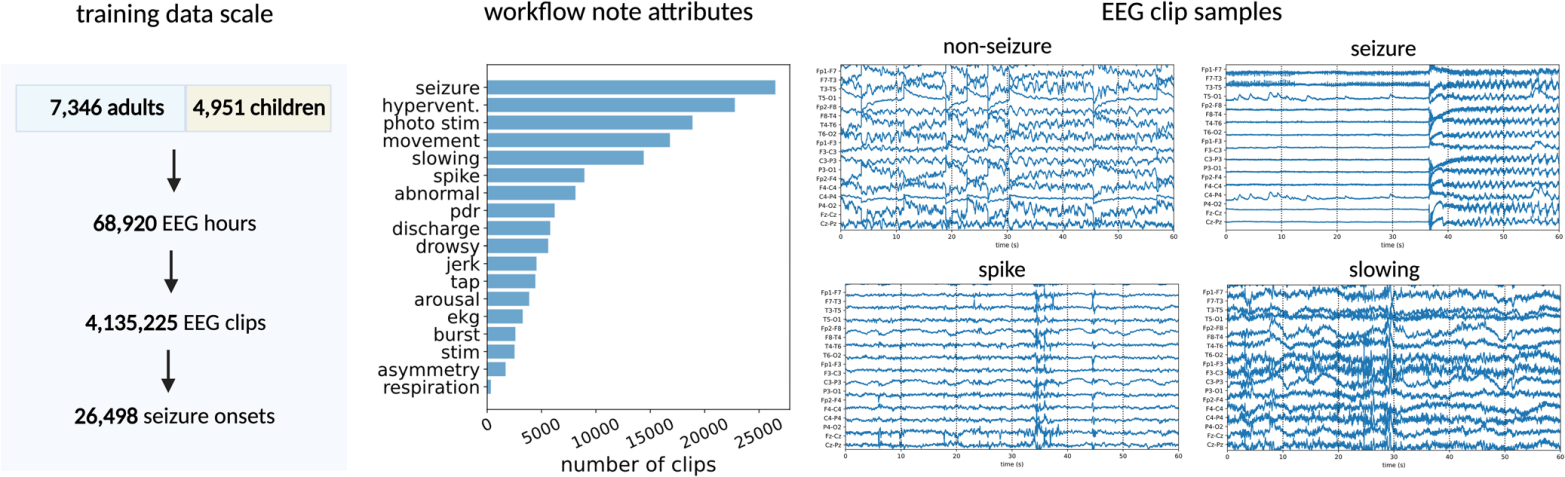
Training dataset overview. In the left panel, we provide statistics on the scale our training dataset of EEG recordings aggregated from adult and pediatric hospitals. In the middle panel, we plot the histogram of attribute labels extracted from workflow notes. In the right panel, we visualize four EEG clips, three of which are non-seizure EEG clips. The non-seizure EEG clips exhibit significant differences in temporal features, motivating the opportunity to use them to increase class specificity.

In clinical routines, experienced technicians predominantly create the workflow notes and are trained to be highly sensitive when flagging potential abnormalities, especially in ambiguous cases. Since each EEG recording may contain multiple seizures, annotators may mark only a subset of seizures, leading to moderate overall seizure recall. Annotations from medical students and fellows, diverse in their experience levels, augment the annotations made by technicians. From manually analyzing the workflow notes, we found 26 recurring event descriptions, or attributes, and wrote simple regular expressions to extract the unique attributes from the workflow notes (e.g., considering synonyms and case-insensitivity). Figure 2 displays a histogram of the 18 most frequent attributes, where for example we have seizure onset annotations for 26,498 EEG clips, spike annotations for 8942 EEG clips, and movement artifact annotations for 16,806 EEG clips.

Given the descriptive nature and high temporal specificity of these workflow notes, marked by precise timestamps indicating the onset of each event, they are a valuable potential resource for supervising ML models. The extensive availability of these notes across both our adult and pediatric hospitals allows for the scalability of training data to unprecedented levels. However, it is pertinent to note that these workflow notes, while extensive, contain false positives and overlooked instances, leading to “noisy” labels—a supervision setting referred to as weak learning^22^. Nevertheless, a study by Saab et al.^5^ demonstrates that the expansive volume of data accessible through workflow notes can compensate for these inaccuracies, facilitating the development of highly proficient EEG ML models. This underscores the statistical principle that, at times, leveraging a larger dataset with inherent noise can be more advantageous in modeling than utilizing a smaller, meticulously hand-labeled dataset, due to the diversity and the variety it offers^23^. In the ensuing experiment, we further validate the assertion that scaling training data with workflow notes greatly benefits the performance of ML models for detecting seizure onset.

We hypothesize that even though workflow notes may contain errors and our regular expressions may extract noisy labels, leveraging workflow notes to scale the training data results in better-performing models compared to training models using a much smaller subset of gold-standard labels. To test our hypothesis, we considered the baseline setting of only having access to our gold-labeled dataset of 37,588 EEG clips. In this baseline setting, we randomly split our gold-standard labeled dataset into train (50%), validation (10%), and test (40%) sets, stratified by patients (i.e., there are no overlapping patients among the three splits). We then trained two classification models, where the first model was trained using the gold-labeled train set (containing 16,058 EEG clips, of which 408 contained a seizure onset), and the second model was trained using the entire training set that was not gold-labeled, resulting in 4,097,637 EEG clips, of which 25,254 contained seizure onset labels extracted from the workflow notes. Details on model architecture and training procedure can be found in the Methods Section. To evaluate seizure onset detection performance, we assessed the Area Under the Receiver Operating Characteristic curve (AUROC) on the held-out test set, and report the 95% confidence intervals.

Leveraging the workflow notes improved the model’s performance, where the model trained on the smaller gold-labeled dataset achieved an AUROC of 73.3 ± 3.2, and the model trained on the much larger workflow-labeled dataset achieved an AUROC of 85.6 ± 0.9.

### Revealing underperforming subgroups

To evaluate whether our models performed less well in certain patient subgroups, we performed a subgroup analysis where we evaluated the change in model performance across multiple clinically relevant subgroups. We carried out the subgroup analysis by using a collection of patient metadata, gold-labeled seizure types, and the attributes from the workflow notes.

For patient subgroups, we recorded whether the patient was from the adult or pediatric hospital, and whether a patient’s EEG recordings were collected in the ICU. For seizure subtypes, we analyzed performance differences among the focal spike-and-wave, evolving rhytmic slowing, and generalized spike-and-wave types (more details in the “Methods” section).

**Fig. 3:**
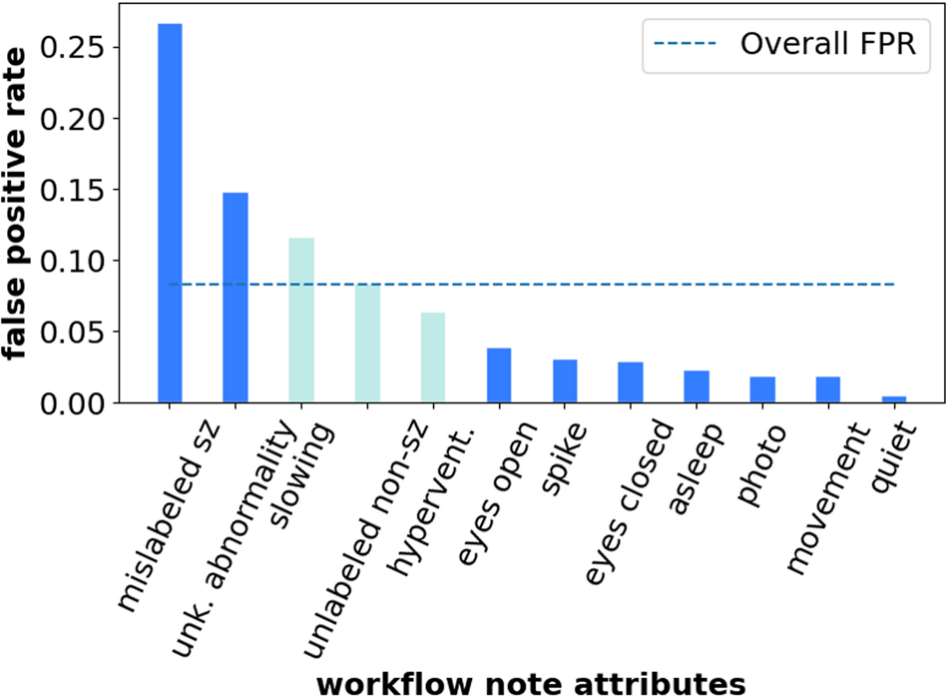
False positive rate among workflow attributes. We plot the FPR with respect to seizure onset for each subgroup within the workflow attributes. Darker shaded bars represent attributes where the FPR is different than the overall FPR with statistical significance using the two-proportion Z-test.

From our subgroup analysis on patient and seizure types in Table 1, we find that our model performed better for patients from the adult hospital with a 6.5 AUROC point difference compared to patients from the pediatric hospital. There were also differences in the performance of the model for various seizure types, with a 5.5 AUROC point difference between focal spike-and-wave and evolving rhythmic slowing seizures. From our subgroup analysis on workflow attributes in Fig. 3, we find that our model had the highest false positive rate (FPR) with respect to seizure onset detection for the “mislabeled sz” attribute (FPR of 0.27), i.e., EEG clips that technicians mislabeled as seizures—a significant difference compared to the overall EEG clips (FPR of 0.08). This is not surprising because the “mislabeled sz” attribute represents EEG clips that technicians thought might be seizures; since the model was trained using technician labels (or workflow notes), the model errors are correlated with the technician errors. We also find from Fig. 3 that the top-3 attributes with the highest FPR ("mislabeled sz”, “unk. abnormality”, and “slowing”) all correspond to non-seizure abnormalities. Details on metrics can be found in the Methods Section.

**Table 1:** Subgroup analysis

	

Model classification performance (AUROC with 95% confidence intervals) for both patient and seizure subgroups. Rows highlighted in blue indicate subgroups that the binary model underperformed on.

### Improving subgroup robustness with class specificity

We hypothesize that our model underperforms on clinically relevant subgroups as a result of the target task being underspecified. Since we train our model to only classify whether an EEG clip contains a seizure onset or not, all abnormal patterns and artifacts are grouped together with normal brain activity patterns (in the non-seizure class). As a result, unlike the training protocols of expert EEG readers, our model does not learn to differentiate among normal activity, abnormal seizure-like activity, and actual seizures, which we hypothesize causes the systematic errors displayed in Table 1 and Fig. 3.

To combat task underspecification, we propose to train a multilabel model, where instead of outputing a binary class (seizure or non-seizure), the model identifies multiple attributes from an EEG clip, such as spikes, slowing, and movement. Importantly, since the workflow notes provide these attributes, we are able to train our multilabel model at no additional annotation cost, and training the model to recognize the additional attributes provides class specificity that we hypothesize can improve model performance. To test our hypothesis, we compared the overall and subgroup performances of a model supervised with binary seizure/non-seizure labels, which we will refer to as the binary model, to the same model trained on the same data but trained to classify the 26 attributes (including seizure onset) extracted from workflow notes, which we will refer to as the multilabel model. While the multilabel model outputs probabilities for all 26 attributes, we only consider the probability of seizure onset for evaluation (a binary classification setting), and calculate the AUROC with respect to the gold-labeled test set for each subgroup.

As shown in Table 2, the multilabel model has significant improvements in both overall performance and subgroup performance (except for 2 of the seizure subgroups). The overall performance improved by 5.9 AUROC points, while the performance on patients from the pediatric hospital improved by 8.3 points, and 7.7 AUROC points for focal spike-and-wave seizure types. Importantly, the improvements in performance significantly minimized the gaps in performance among subgroups. In addition, we compared the FPRs for each attribute (shown in Supplementary Fig. 1) and found that the overall FPR decreased from 0.08 to 0.02. The top 3 attributes with the highest FPR, which correspond to abnormal attributes (mislabeled seizure, unknown abnormality, and slowing), all decreased significantly (e.g., FPR for EEG clips with unknown abnormal patterns decreased from 0.15 to 0.08). We further compared the 2D projected embeddings of the binary and multilabel models in Supplementary Fig. 2, which shows that the embeddings of the multilabel model of abnormal EEG clips cluster more tightly than the embeddings of the binary model, reaffirming that the multilabel model can better differentiate EEG abnormalities.

**Table 2:** Improving subgroup robustness with class specificity

	

Increasing class specificity improves overall model performance along with robustness to hidden subgroups. We stratified our evaluation set by patient and seizure subgroups, where the patient subgroups included patients from the adult hospital, pediatric hospital, or adults within or outside the ICU. We report the average AUROC along with 95% confidence intervals. Rows highlighted in blue indicate subgroups that the binary model underperformed on. We estimated the *p*-value using the DeLong test, which evaluates how statistically significant the improvements of the multilabel model are compared to the binary model.

We also investigated the impact of training a multilabel model on different subsets of the workflow attributes on subgroup robustness. We choose two additional subsets of classes: classifying seizures along with two abnormalities highly relevant to seizures (spikes and slowing), and classifying seizures along with only abnormal attributes (i.e., we remove the following attributes: drowsy, jerk, tap, respiration, eyes open/closed, asleep, ekg, arousal). As shown in Supplementary Table 2, we first found that all multilabel models improved overall seizure detection performance over the binary model. Interestingly, training a multilabel model for detecting seizure onset along with only abnormal attributes performed similarly to the multilabel model trained on all attributes, indicating that increasing class specificity with the abnormal attributes is the most important.

### Measuring clinical utility

A major barrier for technicians and neurophysiologists who have access to commercial seizure detection models is the high number of false alarms^13,24^, which results in alarm fatigue and in clinicians not utilizing model predictions. Therefore, a good metric to assess clinical utility is the average number of false positives after scanning 24 h of EEG (FPs/24 h). In particular, we look at two parameters that directly impact the number of false positives:

- Recall (or sensitivity): Specifying the desired recall implicitly determines the threshold used to binarize the seizure probabilities. While having a higher desired recall is advantageous (since we miss fewer seizures), it is in direct tension with false positives, where number of false positives increase as we increase recall. In some settings, such as counting the precise number of occurring seizures, it may be critical to have a high recall. While in other settings, where the model is used as an assistant to prioritize which parts of the EEG to read first, having a high recall is not as critical. For these reasons, we look at the FPs/24hr for a recall of 0.5, 0.8, and 0.9.
- Delay tolerance (Δ_*t*_): we define the delay tolerance to be the maximum amount of time allowed between the actual seizure onset and the predicted seizure onset. In other words, if the time between actual and predicted seizure onset (*T*) is greater than Δ_*t*_, we count the predicted seizure as a false positive; however, if *T* < Δ_*t*_ then we count the predicted seizure as a true positive. The delay tolerance is an important parameter because not only does it impact how we determine the difference between a true or false positive, but it is also implicitly related to seizure detection latency—the speed in which our model flags seizures. Seizure detection latency may be critical in some settings, for example, if we would like to precisely localize the seizure onset region for patients in the epilepsy monitoring unit, it is critical we accurately analyze the EEG near the true onset zone before spreading occurs. In other settings, such as counting number of seizures, seizure detection latency is not a critical parameter. For these reasons, we look at the FPs/24 h for a delay tolerance of 1 min and 5 min.

**Fig. 4:**
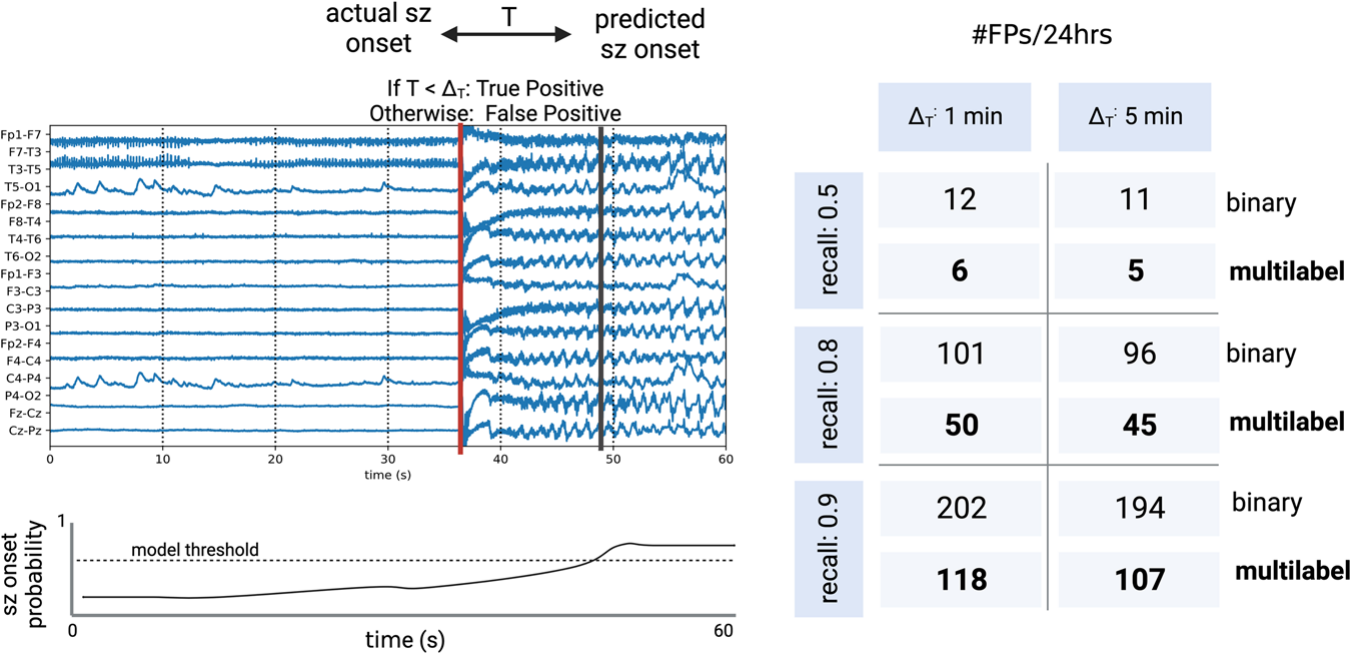
Clinical utility metric. On the left, is an EEG in which the red line indicates the actual seizure (sz) onset, and the black line indicates the predicted seizure onset by the model. The time elapsed between the actual and predicted onset is T, which is used to decide whether the predicted seizure onset is a true positive or false positive (depending on the delay tolerance for the clinical setting). The bottom left plot displays the model seizure onset probabilities across time, where the dashed line indicates the model threshold that is determined by the desired recall value. On the right, we compare the average number of false positives that occur every 24 h of EEG in six different settings: a threshold such that we achieve a recall of 0.5, 0.8, or 0.9, with either of two values of Δ_*T*_, which is a proxy to seizure detection latency (the maximum time between the ground truth and predicted seizure onset we tolerate).

In Fig. 4, we compared the FPs/24 h for six different settings while varying recall and delay tolerance, and observed that the multilabel model improved our clinical utility metric by a factor of roughly 2× across all settings.

## Discussion

In this work, we presented a strategy to improve the trustworthiness of seizure detection models by scaling training data and class specificity in a cost-effective manner. Unlike existing techniques that require fellowship-trained neurophysiologists to annotate thousands of EEGs^11^, we instead leveraged annotations that provide class specificity and are generated in existing clinical workflows^5^, allowing us to scale training data to an unprecedented level of 68,920 EEG hours at no additional annotation cost. In addition to bypassing expert labeling of the training set, workflow notes can also facilitate the ongoing training of healthcare models as additional data are accumulated over time, leading to significant cost savings in terms of upfront and maintenance expenses.

Aside from annotation costs, a major roadblock to successfully deploying healthcare AI is the limited understanding of their errors within hidden subgroups of patients, leading to a lack of trust^12,13,24^. Currently, the gold-standard technique to conduct an in-depth error analysis requires a clinician to manually interpret multiple data samples that the model classified incorrectly and find patterns that combine errors into clinically relevant subgroups. Instead, we proposed to utilize patient metadata, gold-labeled seizure types, and multiple attributes describing EEG events to stratify the evaluation set and analyze differences in model performance. Apart from the gold-labeled seizure types, we are able to extract the attribute labels from the workflow notes, allowing us to greatly improve performance of our model with no additional costs. From our subgroup error analysis, we found that binary seizure classification models may have large performance gaps among patient age groups (−6.5 AUROC points on pediatrics compared to adults), seizure types (−5.5 AUROC points on focal spike-and-wave versus evolving rhythmic slowing), and has significantly higher false positives (+19 FPR points) for non-seizure EEG clips with abnormal brain activity compared to non-seizure clips. Identifying underperforming subgroups is a critical step in our goal towards trustworthy seizure classification models.

Our core hypothesis is that our binary classification model has high false positives on abnormal non-seizure EEG clips as a result of task underspecification. Since fellows are not only trained to differentiate seizure from non-seizure activity, but also to identify multiple artifacts and abnormalities to rule out seizure^25^, we reason that a model should similarly be trained. To reduce high false positive rates and systematic errors, we leveraged attribute labels extracted from workflow notes and trained a multilabel model that learns to classify 26 EEG events such as seizures, spikes, slowing, and movement. We found that such a multilabel model significantly improves overall performance (+5.9 AUROC points), along with closing the performance gap among subgroups, and decreased the false positive rate on abnormal non-seizure clips by 8 FPR points, compared to the binary classification model. We believe this general direction of increasing the specificity of the supervision task is a promising approach to improve model subgroup robustness. Other successful approaches within this direction include increasing spatial specificity for radiology^19^ (e.g., segmentation) and training a chest X-ray model with a comprehensive class ontology^26^.

In our investigation of seizure detection models, we also establish a metric of clinical utility. We report the average number of false positives per 24 h of EEG for different recall and latency settings. We found that across different clinical settings, increasing class specificity reduces the FPs/24 h by a factor of 2×, suggesting that our improvements in subgroup robustness may translate to improvements in clinical utility.

Our proposed supervision strategies for improving trustworthiness of seizure detection models have limitations. First, while workflow notes offer a great alternative to manual expert labeling, the resulting labels come from personnel that are instructed to bias their reading to not miss abnormalities since final diagnosis is reviewed by an interpreting physician, which results in false positive labels and sub-optimal supervision. In addition, our regular expressions to extract labels from the workflow notes may not correctly identify some of the labels, or they may produce errors or not apply to other institutions. Second, while we consider many clinically relevant subgroups, our analysis can be more comprehensive by including many other important groups such as patient demographics, more seizure types, and finer-grained abnormal events. Third, we do not investigate other important robustness settings that include common distribution shifts, such as different EEG devices and patients from multiple hospitals. Other settings for improving trust may also include proper model calibration, calibration scores, and out-of-distribution detection. We believe it is critical to investigate robustness on a comprehensive list of settings before claiming a model to be trustworthy for deployment.

Future work is needed for improving the robustness of seizure detection models. Further scaling training data to include diverse patients can be done by combining our hospital datasets with existing publicly available datasets such as the TUSZ corpus^3,4^. Collaborating with additional hospitals may increase the diversity of our patients, allowing for greater coverage of possible attributes to consider. In a similar spirit, we can utilize publicly available EEG-based models that classify seizures, sleep staging, and brain states^6,27^, to either label relevant attributes or enable transfer learning. Another exciting direction is self-supervised and generative AI, where models do not rely on labeled training data to learn useful data representation. For example, recent work has shown that pretraining to forecast EEG signals boosts performance on rare seizure types^21^. We also envision models that generate text reports from EEG^28^ may prove to have more robust representations due to learning finer-grained concepts.

In summary, our work provides evidence that scaling training data using labels from workflow notes and increasing class specificity are promising techniques to improve robustness of models to detect seizure onset. We believe that combating robustness challenges through in-depth error analyses, and assessing detection performance of models as well as clinical utility metrics, will be critical to continue improving upon the trustworthiness of AI tools for clinical deployment.

## Methods

### Dataset description

Our dataset consists of all EEGs recorded in both the Stanford Hospital and Lucile Packard Children’s Hospital from 2006 to 2017. In total, our dataset contains 68,920 EEG hours from 12,297 patients. Our dataset is diverse, where patients span all ages, come from different hospital locations (ICU, epilepsy monitoring unit, and ambulatory), and have different seizure types and etiologies. More details on the statistics of our diverse patients can be found in Fig. 2 and Supplementary Fig. 2 in Saab et al.^5^.

To prepare input data samples from long-form EEG recordings, we segment each recording into non-overlapping 60-s clips (i.e., stride is 60 s). In total, our dataset contained 4,135,225 clips. To ensure consistent information across patients, we only considered the 19 electrodes from the standard 10–20 International EEG configuration, and exclude premature infants or patients with small heads that prevent the full deployment of the 19 electrodes. We further normalize each EEG clip across the temporal dimension using the global average and standard deviations for each channel. Such normalization of input samples is standard practice in deep learning and we find this improves training.

Two fellowship-trained EEG readers (M.T. and C.L.M.) interpreted a randomly selected subset of EEG recordings, annotating for seizure onset. This resulted in an evaluation set of 37,588 60-s EEG clips (or 626 EEG hours), of which 1244 clips contain seizures from 395 patients. Patients in the evaluation set are excluded from the training set. C.L.M. labeled or supervised the labeling of each EEG clip according to the seizure type as defined by EEG ictal patterns; specifically, whether a seizure was a focal spike-and-wave, evolving rhythmic slowing, generalized spike-and-wave, paroxysmal fast acivity, polyspike-and-wave (myocolonic), or electrographically silent, for a subset of 358 patients from the gold-labeled EEGs. However, due to the low frequency of some seizure types, our evaluations only included focal spike-and-wave, evolving rhytmic slowing, and generalized spike-and-wave types (more details can be found in Supplementary Table 1).

Each EEG recording is complemented by a table of workflow notes, generated from the EEG annotator software utilized by the preliminary annotators, with each row indicating an event description along with the event start time. The event descriptions are free-form text, and while the preliminary annotators use repetitive and standard descriptions, there may be slight deviations. M.T. and C.L.M. analyzed the most common 1000 event descriptions and by consensus determined a set of unique attributes that met two criteria: attributes are (1) visibly detectable on EEGs, and (2) typically used when searching for seizures. For example, attributes of interest included common artifacts that must be distinguished to ascertain seizure onset, such as patient movement, and other abnormalities like spike and slowing. Conversely, event descriptions like “PAT”, which stands for pattern, indicating that the annotator modified the EEG display by altering the arrangement of electrodes, or “IMP”, signifying impedance check, a routine check to verify the proper attachment and conductivity of the electrodes, did not qualify as attributes of interest as they do not meet our two criteria.

From the manual analysis, we identified 26 unique class attributes of interest (listed in Supplementary Table 3). To determine the presence of these attributes in event descriptions, we developed simple regular expressions to recognize various synonyms and acronyms employed by the annotators. For example, an annotator may write “seizure”, “sz”, “spasm”, or “absence”; the description of an unknown abnormality may simply be indicated by “x”, or “xx”; another example is the description of a movement artifact, where the annotator may write “movement”, or “mvt”. Under the guidance of M.T. and C.L.M., we listed all synonyms and acronyms for each attribute to form the regular expressions (refer to Supplementary Table 3).

### Model architecture and training

There have been many deep learning model architectures proposed for seizure classification, such as convolutional models (CNNs)^5,29–31^, recurrent neural networks (RNNs)^32–34^, graphical neural networks (GNNs)^21,35,36^, and more^6,9,37–39^. In our work, we study the impact of training data scale and the specificity of the supervision task on seizure classification performance, and not model architecture. However, due to the inherent advantages of some architectures, such as simplicity and computational efficiency, we chose S4, a recently proposed convolutional-based model motivated by principles in signal processing^40^.

The global architecture of S4 follows a similar deep learning architecture as the transformer encoder, in which each layer is composed of multiple filters, where each filter is a sequence-to-sequence mapping (mixing across time), followed by a non-linear activation function, followed by a linear layer (mixing across filters), and finally a residual connection. The major deviation from the transformer encoder is the sequence-to-sequence filter, which as opposed to an attention mechanism, is a one-dimensional convolutional filter parametrized by linear state-space models (SSMs). An SSM is a fundamental model to represent signals and is ubiquitous across a range of signal processing and control applications^41,42^. A discrete SSM, which maps observed inputs *u*_*k*_ to hidden states *x*_*k*_, before projecting back to observed outputs *y*_*k*_, has the following recurrent form:

1 $${x}_{k+1}={{{\boldsymbol{A}}}}{x}_{k}+{{{\boldsymbol{B}}}}{u}_{k}$$

2 $${y}_{k}={{{\boldsymbol{C}}}}{x}_{k}+{{{\boldsymbol{D}}}}{u}_{k}$$

Where ${{{\boldsymbol{A}}}}\in {{\mathbb{R}}}^{d\times d}$, ${{{\boldsymbol{B}}}}\in {{\mathbb{R}}}^{d\times 1}$, ${{{\boldsymbol{C}}}}\in {{\mathbb{R}}}^{1\times d}$, and ${{{\boldsymbol{D}}}}\in {\mathbb{R}}$ are learnable SSM parameters, and *d* is the dimension of the hidden state *x*. Importantly, we can also compute the SSM as a 1-D convolution, which unlike recurrent models, enables parallelizable inference and training. To see how, if we assume the initial state *x*_0_ = 0, and follow equations (1) and (2), we arrive at the following by induction:

3 $${y}_{k}=\mathop{\sum }\limits_{j=0}^{k-1}{{{\boldsymbol{C}}}}{{{{\boldsymbol{A}}}}}^{k-1-j}{{{\boldsymbol{B}}}}{u}_{j}$$

We can thus compute the output *y*_*k*_ as a 1-D convolution with the following filter:

4 $${{{\boldsymbol{F}}}}=({{{\boldsymbol{C}}}}{{{\boldsymbol{B}}}},{{{\boldsymbol{C}}}}{{{\boldsymbol{A}}}}{{{\boldsymbol{B}}}},{{{\boldsymbol{C}}}}{{{{\boldsymbol{A}}}}}^{2}{{{\boldsymbol{B}}}},\ldots ,{{{\boldsymbol{C}}}}{{{{\boldsymbol{A}}}}}^{\ell -1}{{{\boldsymbol{B}}}})$$

5 $${y}_{k}={({{{\boldsymbol{F}}}}* {{{\boldsymbol{u}}}})}_{k}$$

Following prior work on sequence model classification^40^, we simply use the output squences from the last layer to project from the number of filters to the number of classes (e.g., 2 classes for the binary model and 26 classes for the multilabel model), and perform mean pooling over the temporal dimension before a softmax to output class logits.

There are many advantages of using deep SSMs for long sequence modeling described in recent work^40,43,44^. We highlight the following advantages for EEG modeling: since our EEG clips are of length 12,000, RNNs are slow to train, while CNNs fail to capture long-range dependencies due to limited filter lengths; on the other hand, SSMs are computationally efficient to train (due to their convolutional view), but are also able to capture long-range dependencies with structured initialization of the ***A*** matrix. Moreover, we do not need to learn graph structures among the EEG electrodes, which adds an additional layer of complexity in recent state-of-the-art EEG classification models^6^. Nevertheless, to validate that S4 is a well-suited model architecture for seizure classification, we compared its performance to other architectures on the public TUSZ benchmark in Supplementary Table 4, and found that S4 is competitive with state-of-the-art models while being more computationally efficient.

We trained all models with the cross-entropy loss using the Adam optimizer in Pytorch^45^, with randomly initialized weights. The learning rate was initially set at 0.004 and followed a cosine scheduler^46^. We used a weight decay of 0.1 and a dropout probability of 0.1. Since the training set is very large (~4 million samples) and highly unbalanced with just 0.6% of clips having seizure onset, we used a weighted random sampler with a 25-to-1 bias for positively labeled clips. For more frequent checkpointing, we randomly sampled a maximum of 150,000 clips for each epoch (with replacement), and trained for 200 epochs, while checkpointing on the validation set AUROC. The S4 model architectures had a parameter count of 366k for the binary classification model, and 379k for the multilabel model (due to larger output dimension). The model architecture contained 128 filters per layer for 4 layers with a hidden state dimension *d* of 64, and the gaussian error linear unit for the non-linear activations. We performed a grid search for the initial learning rate, weight decay, and dropout values using our validation set. We used default values for the other hyperparameters, including model architecture.

### Performance metrics

The two main classification metrics used to evaluate seizure classification performance are the the AUROC and the FPR. We chose the classification threshold such that the class balance of the model predictions matches the ground truth class balance. The ROC curve displays the tradeoff between the True Positive Rate (TPR) and FPR for different classification thresholds. Therefore, the AUROC summarizes the ROC curve in a single scalar value regardless of the specific classification threshold chosen. The FPR and TPR are defined as follows:

6 $$FPR=\frac{FP}{FP+TN}$$

7 $$TPR=\frac{TP}{TP+FN}$$

where true-positives (TP) are correct seizure classifications, true-negates (TN) are correct non-seizure classifications, false-positives (FP) are incorrect seizure classifications, and false-negatives (FN) are incorrect non-seizure classifications. To calculate 95% confidence intervals and p-values when comparing the AUROC of two models, we used the DeLong test^47^.

### Reporting summary

Further information on research design is available in the Nature Research Reporting Summary linked to this article.

### Supplementary information

Supplementary Materials

Reporting Summary

## Data Availability

The Stanford clinical datasets used in this study are subject to restrictions regarding the availability of Protected Health Information. They were accessed with approval from the Institutional Review Board solely for the purpose of this specific study and are not accessible to the public.

## Code availability

The code used to generate the main results in this manuscript can be found in the following github repository: https://github.com/khaledsaab/eeg_robustness.
