# Supplementary material for: Towards trustworthy seizure onset detection using workflow notes
Source: NPJ Digit Med. 2024 Feb 21;7:42. doi: 10.1038/s41746-024-01008-9 (PMC10881468; doi:10.1038/s41746-024-01008-9)
Supplement: Supplementary file 1 — Supplementary Materials [file 41746_2024_1008_MOESM1_ESM.pdf]

Supplementary Materials: Towards trustworthy seizure onset detection using workflow notes

Khaled Saab, Siyi Tang, Mohamed Taha,
Christopher Lee-Messer, Christopher Ré, and Daniel L. Rubin

**seizure type**	**seizure count**
focal spike-and-wave	181
evolving rhythmic slowing	81
generalized spike-and-wave	53
polyspike-and-wave (myoclonic)	22
paroxysmal fast activity	17
fast spiking	7
sz without clear electrographic change	2
electrographically silent	1

Supplementary Table 1: **Seizure types:** Seizure counts for different seizure types as defined by EEG ictal patterns in a subset of our gold-labeled evaluation set.

Subgroups	sz only	sz/spikes/slowing	sz / abnormal attributes	sz / all attributes
Overall	85.6 ± 0.9	**88.4 ± 1.0**	**91.4 ± 0.9**	**91.5 ± 0.9**
Adults	89.4 ± 1.1	89.6 ± 1.5	**92.8 ± 1.0**	**92.7 ± 1.1**
Pediatrics	82.9 ± 1.5	**87.8 ± 1.5**	**90.7 ± 1.4**	**91.2 ± 1.3**
Adults outside ICU	9.4 ± 1.7	88.7 ± 2.5	91.1 ± 1.7	**91.7 ± 1.7**
Adults from ICU	88.5 ± 1.2	**91.2 ± 1.5**	**94.7 ± 1.1**	**94.0 ± 1.2**
Focal spike-and-wave	84.3 ± 2.6	**89.4 ± 2.4**	**90.9 ± 2.0**	**92.0 ± 1.7**
Evolving rhythmic slowing	89.8 ± 3.3	91.4 ± 4.1	**94.8 ± 2.3**	93.2 ± 3.0
Generalized spike-and-wave	85.5 ± 4.0	73.7 ± 7.5	89.9 ± 3.5	90.0 ± 4.3

Supplementary Table 2: **Subgroup robustness:** Increasing task specificity improves overall model performance along with robustness to hidden subgroups. Columns increase in class specificity from left to right; e.g., “sz only” indicates a model trained to only detect seizures, while “sz/spikes/slowing” indicates a model trained to detect seizures, along with spikes and slowing abnormalities. We stratify our evaluation set by patient and seizure subgroups, where the patient subgroups include patients from the adult hospital, children hospital, or adults within or outside the ICU. We report the average AUROC for the multilabel seizure detection model along with 95% confidence intervals. Bolded numbers indicate statistically significant lifts over the binary classification model. “sz” stands for seizure.

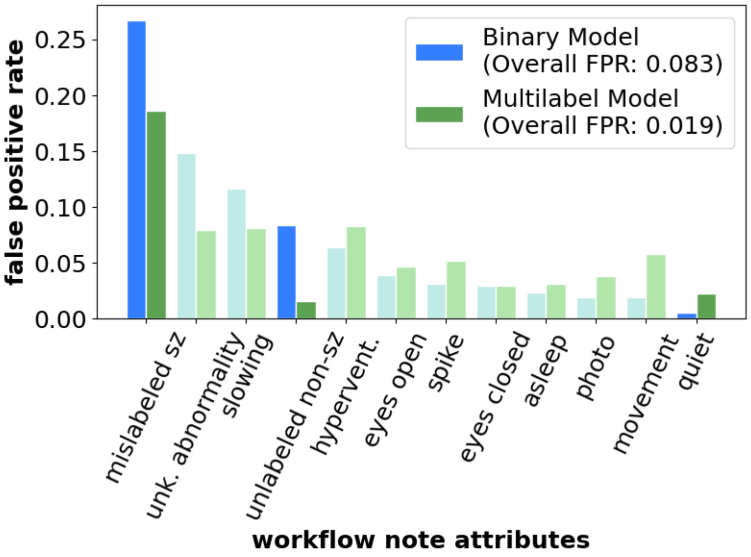

Supplementary Figure 1: **FPR attribute analysis:** False positive rates with respect to seizure detection across subsets of our evaluation set stratified by the workflow attributes for the binary and multilabel model. The seizure detection threshold was chosen such that the class balance of the model predictions matched the ground truth class balance. Darker shaded bars represent attributes where the FPR between the binary and multilabel models are different with statistical significance using the two-proportion Z-test.

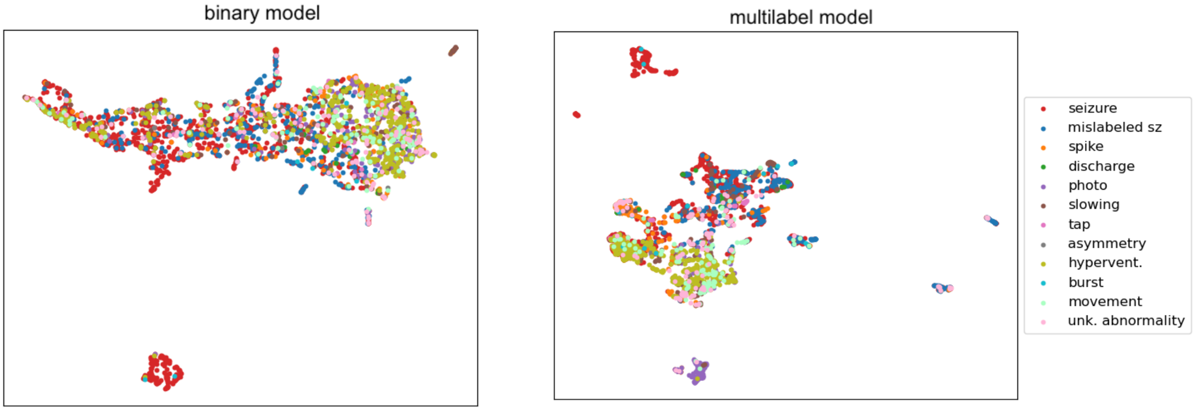

Supplementary Figure 2: **Model embedding analysis:** UMap-projected embeddings show that the multilabel model embeddings cluster the abnormal attributes (mislabeled sz, spike, slowing, unknown abnormality) more tightly compared to the binary model embeddings, reaffirming that the multilabel model has learned to more effectively differentiate seizures from other EEG abnormalities.

**attribute**	**regular expression**
seizure	“seizure | sz | absence | spasm”
spike	“spike”
slowing	“slow”
photoelectric stimulation	“photo”
stimulation	“stim”
posterior dominant rhythm	“pdr”
unknown abnormality	“ˆx*$”
movement artifact	“movement | mvt”
EKG artifact	“ekg”
discharge	“discharge | discharges”
tapping artifact	“tap”
hyperventilation	“hv”
jerking	“jerk”
drowsy	“drowsy”
asymmetry	“asymmetry”
arousal	“arousal”
respiration	“respiration”
asleep	“asleep | sleep”
awake	“awake”
burst	“burst”
quiet	“quiet”
suspicion in left hemisphere	“ˆL*$”
suspicion in right hemisphere	“ˆR*$”
eyes closed	“eyes closed”
eyes opened	“eyes opened”

Supplementary Table 3: **EEG attributes and regular expressions to extract EEG attributes from workflow notes:** For each regular expression, we turn off the case sensitivity flag.

Model	LSTM	CNN-LSTM	Dense-CNN	DCRNN	Graphs4mer	S4
TUH	71.5 ± 1.6	68.2 ± 0.3	79.6 ± 1.4	80.4 ± 1.5	90.6 ± 1.2	87.7 ± 1.1

Supplementary Table 4: Architecture comparisons on TUH v1.5.2[1] test set (AUROC). Our chosen architecture (S4) is competitive with SoTA seizure detection methods. Performance of baseline models (first five columns) are taken from Tang et al., 2022[2].

References

1. Shah, V. *et al.* The temple university hospital seizure detection corpus. *Frontiers in neuroinformatics* **12**, 83 (2018).

2. Tang, S. *et al.* Spatiotemporal modeling of multivariate signals with graph neural networks and structured state space models. *arXiv preprint arXiv:2211.11176* (2022).
